# Serum levels of matrix metalloproteinases 1, 2, and 7, and their tissue inhibitors 1, 2, 3, and 4 in polytraumatized patients: Time trajectories, correlations, and their ability to predict mortality

**DOI:** 10.1371/journal.pone.0300258

**Published:** 2024-03-08

**Authors:** Lukas L. Negrin, Greta L. Carlin, Robin Ristl, Stefan Hajdu

**Affiliations:** 1 University Department of Orthopedics and Trauma Surgery, Medical University of Vienna, Vienna, Austria; 2 University Department of Obstetrics and Gynecology, Medical University of Vienna, Vienna, Austria; 3 Center for Medical Statistics, Informatics and Intelligent Systems, Medical University of Vienna, Vienna, Austria; University of Vermont College of Medicine, UNITED STATES

## Abstract

There has been limited research on assessing metalloproteinases (MMPs) 1, 2, and 7, as well as their tissue inhibitors (TIMPs) 1, 2, 3, and 4 in the context of polytrauma. These proteins play crucial roles in various physiological and pathological processes and could be a reliable tool in polytrauma care. We aimed to determine their clinical relevance. We assessed 24 blunt polytrauma survivors and 12 fatalities (mean age, 44.2 years, mean ISS, 45) who were directly admitted to our Level I trauma center and spent at least one night in the intensive care unit. We measured serum levels of the selected proteins on admission (day 0) and days 1, 3, 5, 7, and 10. The serum levels of the seven proteins varied considerably among individuals, resulting in similar median trend curves for TIMP1 and TIMP4 and for MMP1, MMP2, TIMP2, and TIMP3. We also found a significant interrelationship between the MMP2, TIMP2, and TIMP3 levels at the same measurement points. Furthermore, we calculated significant cross-correlations between MMP7 and MMP1, TIMP1 and MMP7, TIMP3 and MMP1, TIMP3 and MMP2, and TIMP4 and TIMP3 and an almost significant correlation between MMP7 and TIMP1 for a two-day-lag. The autocorrelation coefficient reached statistical significance for MMP1 and TIMP3. Finally, lower TIMP1 serum levels were associated with in-hospital mortality upon admission. The causal effects and interrelationships between selected proteins might provide new insights into the interactions of MMPs and TIMPs. Identifying the underlying causes might help develop personalized therapies for patients with multiple injuries. Administering recombinant TIMP1 or increasing endogenous production could improve outcomes for those with multiple injuries. However, before justifying further investigations into basic research and clinical relevance, our findings must be validated in a multicenter study using independent cohorts to account for clinical and biological variability.

## Introduction

Polytraumatized patients are among the most vulnerable in trauma emergencies. Since the combination of multiple injuries outweighs the sum of the individual damages, polytrauma victims represent the ultimate challenge in trauma care. Due to improvements in prehospital and clinical management through standardized, priority-oriented algorithms and medical-technical progress, the mortality rate of polytraumatized patients admitted to the intensive care unit has continuously decreased over the last decades [1]. Thus, whether polytraumatized patients survive is increasingly receding into the background, while the question of how they survive is becoming increasingly important. Survivors often suffer long-term sequelae (physical functional limitations, neurological deficits, chronic pain, psychological complaints, and cognitive disorders) that result in restrictions on daily life and often prevent a return to work. The pathophysiology after polytrauma is highly complex, and many different pathways are activated straightaway in a parallel fashion [2]. A better understanding of the underlying mechanisms on the molecular, cellular, and humoral levels is crucial for a more precise prediction of complications resulting in the in-time choice of the adequate treatment regimen. The significant heterogeneity in polytrauma victims, rooted in myriad etiologies and injury combinations, complicates the search for substantial biomarkers in any setting, i.e., regardless of the concomitant injuries present and the inclusion and exclusion criteria selected. Pilot studies that strive to identify clinically relevant biomarker candidates are based on the trial-and-error principle to a large extent, thus not surprisingly leading to positive and negative results in their findings.

Matrix metalloproteinases (MMPs) are proteolytic enzymes with similar functional domains [3], being members of the metzincin protease superfamily of zinc-dependent endopeptidases [4]. Of the 28 types of MMPs identified in vertebrates, at least 24 are expressed in human tissue [5]. These enzymes can degrade almost every component of the extracellular matrix (ECM) [6]. Thus, they are involved in the invasion and metastasis of many types of cancer [7]. Moreover, they regulate inflammation, epithelial-mesenchymal transition, cell proliferation, angiogenesis, and apoptosis [8]. Based on structure and substrate specificity, MMPs include six groups: collagenases, gelatinases, stromelysins, matrilysins, membrane-type MMPs, and other non-classified MMPs [9].

MMP1 (collagenase-1) particularly cleaves interstitial collagens I, II, and III into characteristic 3/4 and 1/4 fragments [10, 11]. Moreover, it degrades other extracellular components like, for example, proteoglycans or structural proteins, therefore enabling cell migration or the release of active molecules from ECM stores [12]. MMP1 is secreted by various cells, including fibroblasts, keratinocytes, endothelial cells, macrophages, and hepatocytes [5, 11]. It plays a role in leukocyte migration and vascular dysfunction [13]. Increased levels have been detected in slow-to-heal and venous wounds [14] and inflammatory conditions [15]. MMP2 (Gelatinase-A), the most abundant MMP [16], is named for its ability to degrade gelatin [17] but also digests collagens, including I, II, III, IV, VII, and X [18]. It is released among other cells by fibroblasts, keratinocytes, endothelial cells, chondrocytes, osteoblasts, leukocytes, platelets [5], and epithelial cells [19] and is implicated in the differentiation, regeneration, and repair of skeletal muscles [20]. Increased levels of MMP2 were detected in the serum of patients suffering from chronic kidney disease [21]. MMP7, known as matrilysin, is the smallest MMP [22]. It is expressed by epithelial cells, keratinocytes, fibroblasts, and macrophages [23] and plays an essential role in activating immune mechanisms [23] in organs such as the lungs and intestines [23]. Its substrates are fibronectin, laminin, collagen IV, and gelatin [5].

Like most MMPs, MMP1, MMP2, and MMP7 are synthesized and secreted as inactive zymogens [24–26], in which a propeptide domain blocks the active site [27] requiring proteolytic cleavage under physiological conditions to promote the release of the propeptide domain bound to the catalytic site and generate active MMPs [28]. The proteolytic activity of MMPs during tissue remodeling is mainly regulated by four endogenous proteins known as tissue inhibitors of metalloproteinases TIMP1, TIMP2, TIMP3, and TIMP4 [29, 30], which bind tightly to the MMP active site [31], forming 1:1 stoichiometric complexes [32]. In general, all TIMPs can inhibit all known MMPs; however, the efficacy of MMP inhibition varies with each TIMP [33]. Although the TIMPs share many fundamental similarities, they show distinctive structural features, biochemical characteristics, and expression patterns [30]. TIMPs are soluble in the ECM, except for TIMP3, which is bound to the ECM [32]. TIMP1 is synthesized by keratinocytes, fibroblasts, smooth muscle cells, and endothelial cells [34]. TIMP2 is secreted by macrophages [35], epithelial cells [36], fibroblasts [37], and keratinocytes [38]. TIMP3 is released by endothelial cells, keratinocytes [38], epithelial cells [39], fibroblasts [34] and mesenchymal stem cells [40], and TIMP4 by pericytes [41], adipocytes [42], epithelial cells, and mesenchymal cells [43].

Extensive research has been conducted on the involvement of MMP1, MMP2, MMP7, and the four TIMPs in cancer progression, migration, and metastasis [6, 32, 44, 45]. Moreover, the serum or plasma levels of these proteins have been investigated in a wide range of diseases, including geriatric osteoporosis (MMP2, TIMP1, TIMP2 [46]), musculoskeletal diseases (MMP1, MMP 2, TIMP1 [47]), vasculitis (MMP1 [48]; MMP2, TIMP1, TIMP 2 [49]), coronary artery disease (MMP1, [50]), cardiovascular disease (MMP1, TIMP1, TIMP2 [51]), kidney injury (TIMP2 [52]), diabetic kidney disease (MMP7 [53]), tuberculosis (MMP1, TIMP1, TIMP2, TIMP3, TIMP4 [54]; MMP7, TIMP 4 [55]), interstitial lung disease (MMP1, TIMP2, TIMP3 [56]), pulmonary hypertension (MMP2 [57]), chronic hepatitis B virus infection (MMP2 [58]), transthyretin amyloidosis (MMP1 [59]), leishmaniasis (MMP2 [60]), and seizures (TIMP1 [61]). Moreover, the balance between MMP and TIMP levels has been shown to be important. As a result, studies have been concentrating on the ratios of MMPs to TIMPs [46, 62–66].

In trauma patients, however, the selected proteins are only poorly explored. According to an immunohistochemical investigation in post-mortem human samples, MMP1 and MMP2 are mainly upregulated in the first weeks after spinal cord injury [67]. In patients suffering from traumatic brain injury (TBI), MMP7 serum levels are a diagnostic marker of blood-brain barrier dysfunction [68], and TIMP1 serum levels predict mortality [69, 70]. Increased plasma levels of TIMP3 are associated with a higher risk of contracting acute respiratory distress syndrome (ARDS) and dying after severe isolated TBI [71]. Elevated levels of MMP2 were detected in the plasma of patients suffering moderate or severe TBI [72]. Finally, TIMP1 serum levels were significantly higher in older (≥ 60 years) compared to younger polytrauma victims (< 60 years) [73] and in patients who received massive blood transfusions compared to those who did not [74].

Given their involvement in various biological processes and the results already presented in the literature, we hypothesized that serum levels of MMP1, MMP2, and MMP7 and their four inhibitors TIMP1, TIMP2, TIMP3, and TIMP4 might be of clinical value in polytrauma care. Thus, we aimed to (1) assess the trend curves of the serum levels of the seven selected proteins within ten days, (2) highlight similarities in their temporal courses, (3) search for correlations between any two serum levels, (4) examine the informative significance of serum level ratios, and (5) elucidate whether serum levels determined at admission are predictive biomarker candidates for in-hospital mortality.

## Patients and methods

### Patients

We conducted a pilot study from January 1 to December 31, 2019, involving 36 consecutive patients who had suffered blunt trauma and were aged 18 years or older. These patients had an Injury Severity Score (ISS) of 16 or higher and did not have any chronic inflammatory lung diseases or malignancies. All of them were transported directly from the accident site to our Level I trauma center and stayed at least one night in the intensive care unit. The control group formed ten healthy adults who had responded to our call for volunteers.

### Protein assessment

As part of the routine withdrawal, blood was taken from each polytraumatized patient during the initial examination at admission (day 0) and throughout hospitalization on days 1, 3, 5, 7, and 10, using one separation gel tube (Vacuette R© 8 mL; Greiner Bio-One International) every time. Participants in the control group only underwent a single blood draw. Shortly after sampling, the serum was extracted by centrifuging the blood at 3000 × g for 15 min at room temperature; it was isolated and subsequently stored at -80°C until assayed. We used R&D Systems® “Human Magnetic Luminex® Performance Assay MMP Base Kit LMPM000” and “Human TIMP Multiplex Kit LKT003” for assessing the serum protein levels.

### Ethical statement

The Medical University of Vienna (Austria) institutional review board approved this study under vote number 1617/2018. The study was conducted in accordance with the Declaration of Helsinki and local regulations. All patients provided written informed consent.

According to our study protocol that was submitted to and accepted by the local Ethics Committee we generally informed our patients about blood sampling as soon as possible. We assessed their capacity to provide consent one to two hours later by verifying if they comprehended that their involvement in the study simply required an additional tube of blood to be drawn as part of routine blood sampling, and not any medical treatment. If patients refused written consent, we did not withdraw further blood samples and destroyed the previously sampled material at their request.

### Statistical analysis

We analyzed statistics using the software R 3.5. and IBM SPSS Statistics 29. We presented demographic data by mean and standard deviation and characterized protein levels by median and range, whereas we displayed categorical data in absolute frequencies and percentages. We applied Mann-Whitney-U-tests for comparisons between independent groups and the Wilcoxon signed rank tests to contrast protein levels within a patient between time points. We computed Spearman’s correlation coefficients to reveal associations between protein levels at the same measurement points. To analyze the common intra-individual association for paired repeated measures according to Bland and Altman we calculated the correlation coefficients with repeated measurements [75]. To evaluate whether protein levels may predict the death of an injured patient, we conducted univariable binary logistic regression analyses for in-hospital mortality, with every protein level assessed at admission as the predictor. Odds ratios (OR) are displayed with 95% confidence intervals (CI). The receiver operating characteristic (ROC) curve was plotted for graphical analysis, providing the area under the curve (AUC) and its 95% CI. Finally, we defined the cutoff level by the maximum sum of sensitivity and specificity [76]. A p-value less than 0.05 was generally considered statistically significant.

## Results

### Clinical course

Our study group included 23 males and 13 females; one-third (six males and six females) died during their hospital stay (four patients on day 1, three on day 4, and one on days 2, 3, 7, 11, and 42, respectively). Mechanisms of injury included traffic accidents (12), pedestrian hits by vehicles (5) and an oncoming subway (1), falls from height (6), hit by a fallen tree branch (1), committed (3) or attempted (4) suicide by jumping, and attempted suicide by throwing themselves in front of a train (4). Overall baseline characteristics are shown in Table 1.

**Table 1:** Baseline characteristics of the study group. Displayed values are mean ± standard deviation or median [minimum−maximum] or absolute (relative) frequencies.

Age (years)		44.2 ± 22.1
ISS		43 ± 16
AIS_Head_		3.5 [0−5]
AIS_Face_		0 [0−3]
AIS_Chest_		4 [0−5]
AIS_Abdomen_		2 [0−5]
AIS_Extremitis_		3 [0−5]
AIS_External_		1 [0−2]
Complications	Sepsis (n)	3 (8.3%)
	ARDS (n)	8 (22.2%)
	Pneumonia (n)	10 (27.8%)
	Acute kidney injury (n)	5 (13.9%)
	Hemofiltration (n)	3 (8.3%)
	Urinary tract infection (n)	3 (8.3%)
	Pancreatitis (n)	2 (5.6%)
	Clostridium difficile infection (n)	1 (2.8%)
Protein levels day 0 (pg/mL)	MMP1	2810 [707−7281]
	MMP2	226150 [85526−692573]
	MMP7	1870 [911−19534]
	TIMP1	178664 [55271−394953]
	TIMP2	98276 [56406−163617]
	TIMP3	22675 [6078−40467]
	TIMP4	1767 [588−5565]
Reference levels (pg/mL)	MMP1	4360 [1547−12181]
	MMP2	287229 [132174−421060]
	MMP7	1328 [699−1819]
	TIMP1	156597 [81393−193735]
	TIMP2	107452 [75496−130669]
	TIMP3	19936 [10976−44674]
	TIMP4	1389 [441−2270]

ISS, Injury Severity Score; AIS, Abbreviated Injury Scale; ARDS, Acute Respiratory Distress Syndrome.

Surviving patients had a lower mean age (38.3 ± 19.0 versus 56.2 ± 20.7 years; p = 0.007), lower mean ISS (37 ± 14 versus 56 ± 13), and lower percentages of severe head injury (AIS_Head_ ≥ 3; 41.7% versus 83.3%; p = 0.018) and lower percentages of severe extremity injury (AIS_Extremity_ ≥ 3; 58.3% versus 91.7%; p = 0.041) than fatal cases. No further significant demographic differences were found between survivors and fatalities.

### Time trajectories

**Fig 1:**
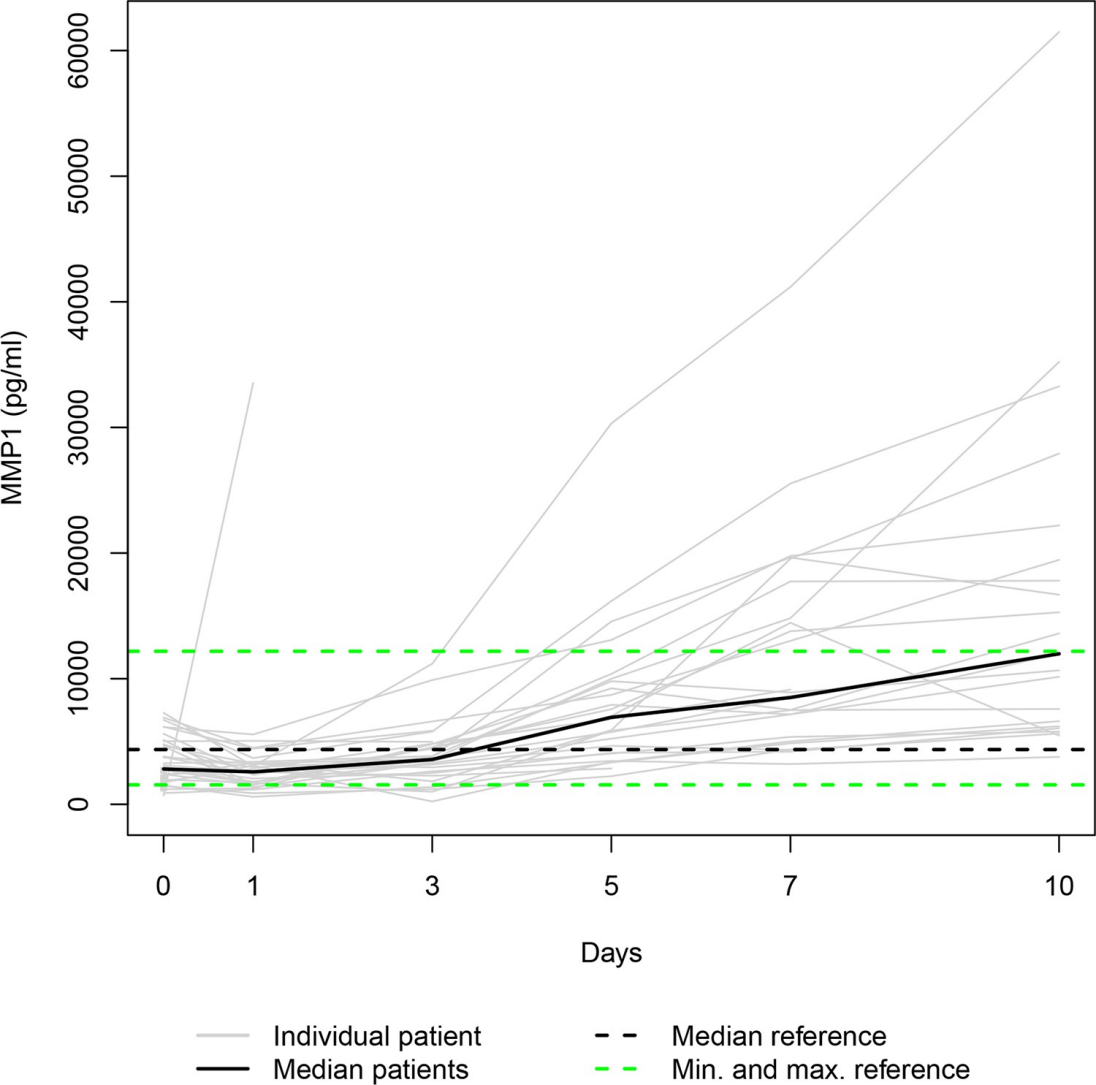
MMP1 serum levels. In the study group, individual MMP1 serum levels (gray lines) and median serum MMP1 level (bold black line). Median (dashed black line), minimum, and maximum (dashed green lines) MMP1 levels in the healthy volunteers.

**Fig 2:**
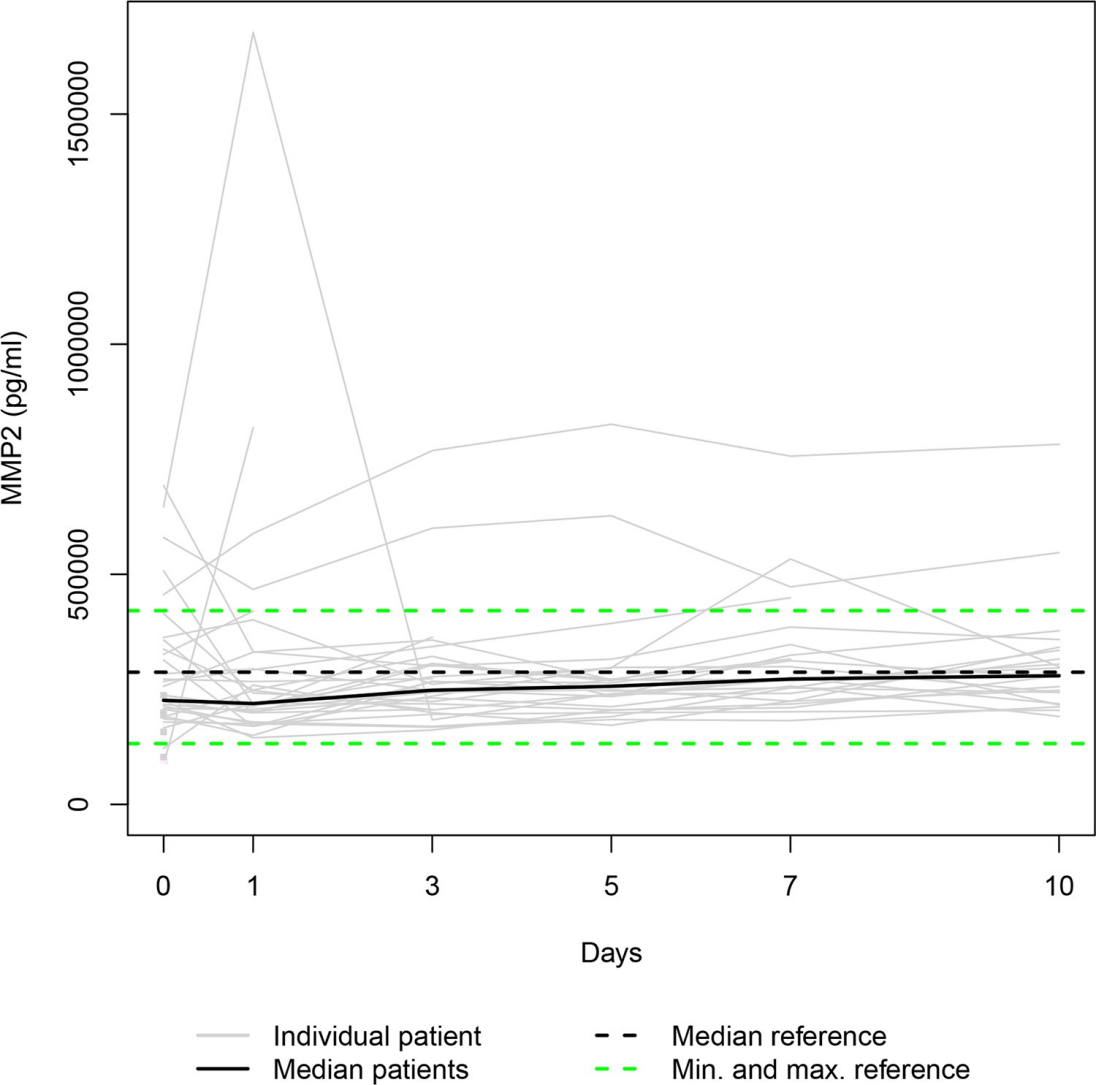
MMP2 serum levels. In the study group, individual MMP2 serum levels (gray lines) and median serum MMP2 level (bold black line). Median (dashed black line), minimum, and maximum (dashed green lines) MMP2 levels in the healthy volunteers.

**Fig 3:**
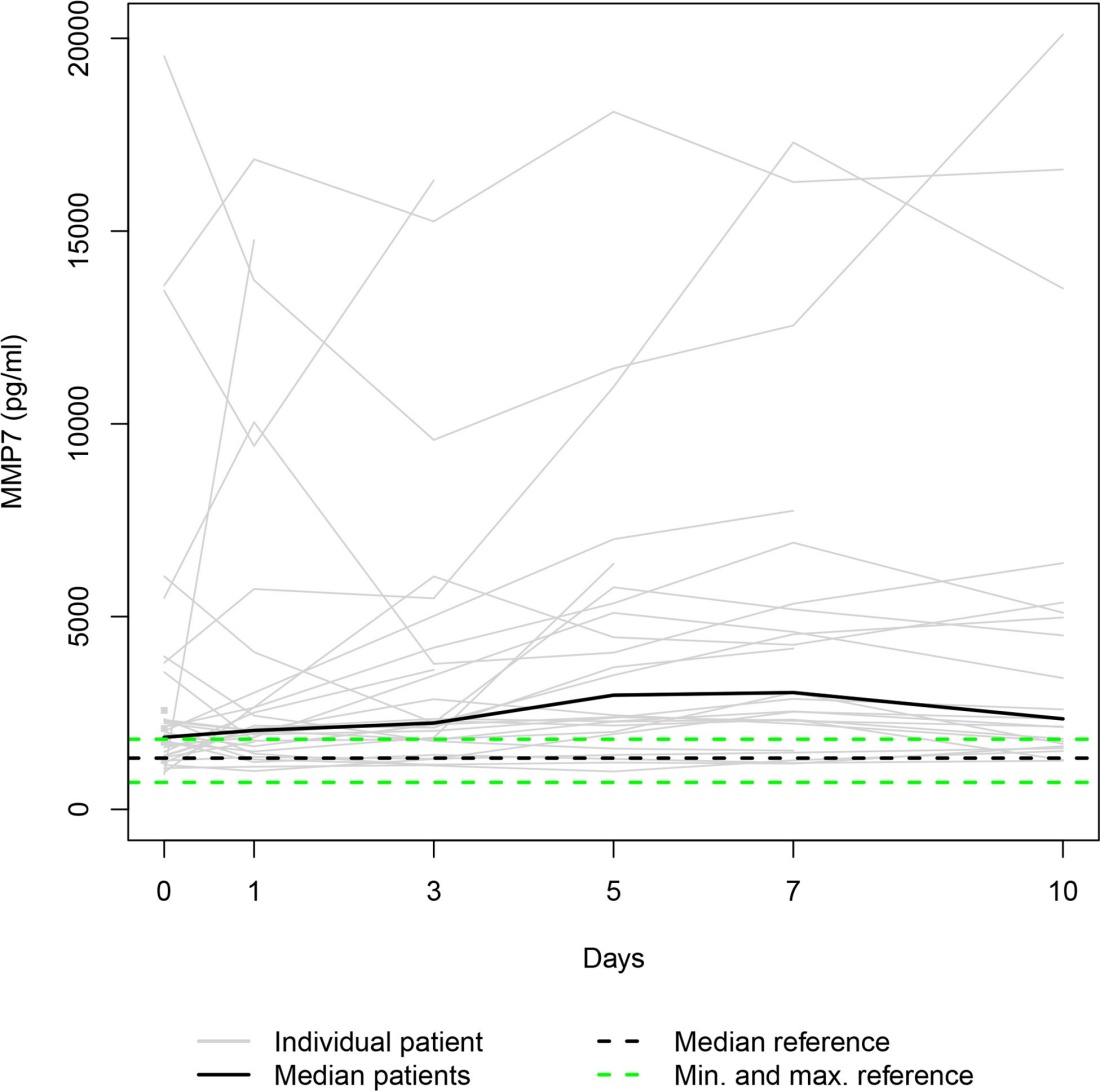
MMP7 serum levels. In the study group, individual MMP7 serum levels (gray lines) and median serum MMP7 level (bold black line). Median (dashed black line), minimum, and maximum (dashed green lines) MMP7 levels in the healthy volunteers.

**Fig 4:**
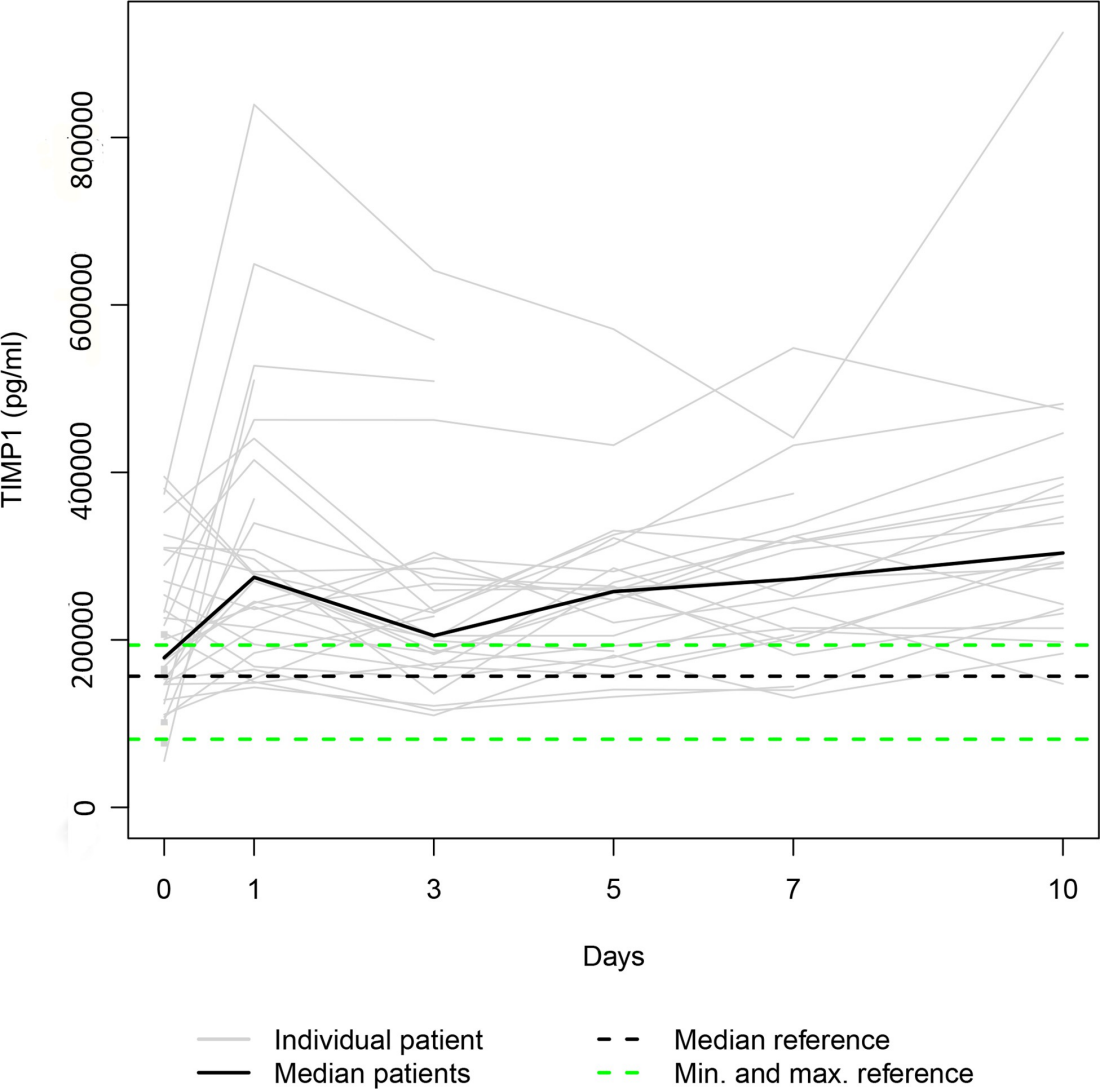
TIMP1 serum levels. In the study group, individual TIMP1 serum levels (gray lines) and median serum TIMP1 level (bold black line). Median (dashed black line), minimum, and maximum (dashed green lines) TIMP1 levels in the healthy volunteers.

**Fig 5:**
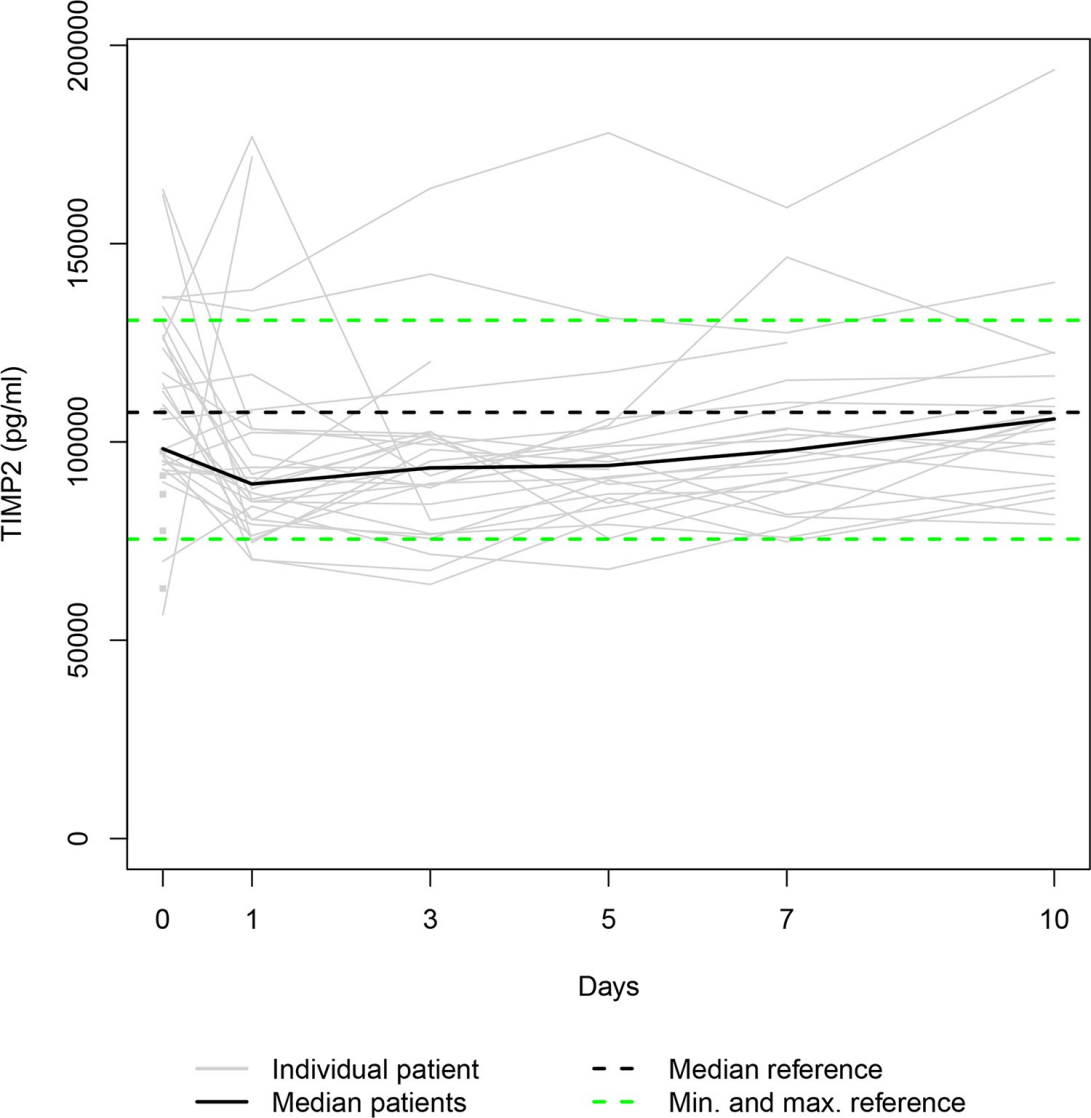
TIMP2 serum levels. In the study group, individual TIMP2 serum levels (gray lines) and median serum TIMP2 level (bold black line). Median (dashed black line), minimum, and maximum (dashed green lines) TIMP2 levels in the healthy volunteers.

**Fig 6:**
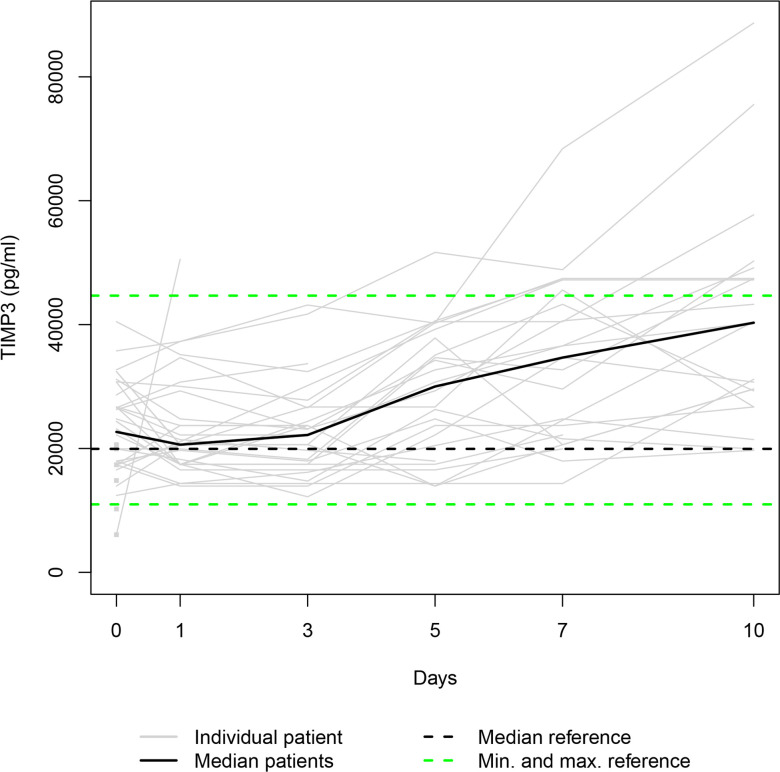
TIMP3 serum levels. In the study group, individual TIMP3 serum levels (gray lines) and median serum TIMP3 level (bold black line). Median (dashed black line), minimum, and maximum (dashed green lines) TIMP3 levels in the healthy volunteers.

**Fig 7:**
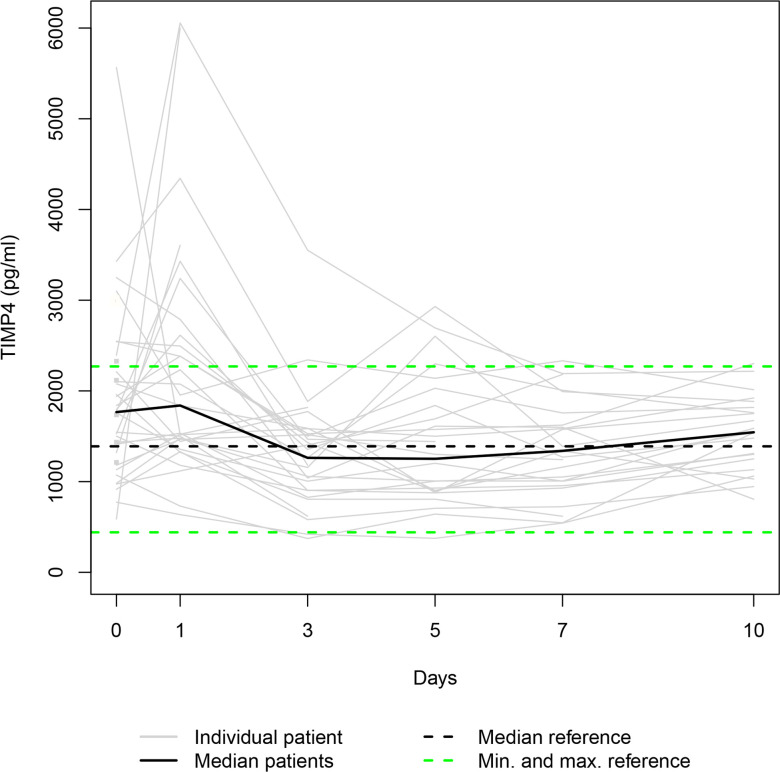
TIMP4 serum levels. In the study group, individual TIMP4 serum levels (gray lines) and median serum TIMP4 level (bold black line). Median (dashed black line), minimum, and maximum (dashed green lines) TIMP4 levels in the healthy volunteers.

Our data set was solely complete for day 0 since ten patients died during the study period, and three patients refused consent for further blood draws when they attained consciousness on days 1, 5 and 7, respectively. Thus, 36, 31, 28, 25, 24, and 23 samples were available for serum level assessment on days 0, 1, 3, 5, 7, and 10. The Individual and the median serum levels of the selected proteins are presented in Figs 1–7, which also display the corresponding median and range in the healthy control group.

The median levels of MMP1, MMP2, TIMP2, and TIMP3 declined between the measurements on days 0 and 1 (p = 0.003; n.s.; p = 0.007; n.s.) and increased between days 1 and 10 (p<0.001; p = 0.012; p = 0.001; p<0.001). The four median day 10 levels were higher than the corresponding median day 0 level. However, only the difference between the MMP1 day 0 and day 10 levels and the TIMP3 day 0 and day 10 levels reached statistical significance (p<0.010; p<0.001). In contrast, the median levels of TIMP1 and TIMP4 rose from day 0 to day 1 (p = 0.001; n.s.), fell between days 1 and day 3 (p = 0.002; p<0.001), and rose between days 3 and 10 (p<0.001; p = 0.033) to a significantly higher/lower level (p = 0.004; p = 0.011) than at the time of admission. Lastly, the median MMP7 level increased between admission and day 7 (n.s.) and decreased between days 7 and 10 (n.s.), resulting in a higher median day 10 level than the median day 0 level (n.s.).

### Spearman correlations

The Spearman correlation coefficients were calculated to determine the interrelationship of serum levels at the same measurement points. Surprisingly, significant coefficients were only calculated between the MMP2, TIMP2, and TIMP3 levels. They are presented in Table 2.

**Table 2:** Spearman correlations between protein serum levels.

	MMP2 day 0	TIMP2 day 0	TIMP3 day 0
MMP2 day 0	1	0.796**	0.632**
TIMP2 day 0	0.796**	1	0.789**
TIMP3 day 0	0.632**	0.789**	1
	MMP2 day 1	TIMP2 day 1	TIMP3 day 1
MMP2 day 1	1	0.826**	0.526**
TIMP2 day 1	0.826**	1	0.572**
TIMP3 day 1	0.526**	0.572**	1
	MMP2 day 3	TIMP2 day 3	TIMP3 day 3
MMP2 day 3	1	0.681**	0.418*
TIMP2 day 3	0. 681**	1	0.631**
TIMP3 day 3	0.418*	0.631**	1
	MMP2 day 5	TIMP2 day 5	TIMP3 day 5
MMP2 day 5	1	0.756**	0.469*
TIMP2 day 5	0.756**	1	0.676**
TIMP3 day 5	0.469*	0.676**	1
	MMP2 day 7	TIMP2 day 7	TIMP3 day 7
MMP2 day 7	1	0.731**	0.523*
TIMP2 day 7	0.731**	1	0.735**
TIMP3 day 7	0.523*	0.735**	1
	MMP2 day 10	TIMP2 day 10	TIMP3 day 10
MMP2 day 10	1	0.673**	0.691**
TIMP2 day 10	0.673**	1	0.760**
TIMP3 day 10	0.691**	0.760**	1

* The correlation is significant at a level of 0.05 (both sides)

** The correlation is significant at a level of 0.01 (both sides)

In our study, we found significant correlations between the ISS and the MMP1 levels on day 0 (r = -0.416), and the TIMP1 levels on day 1 (r = 0.646), day 3 (r = 0.418), day 5 (r = 0.444), and day 7 (r = 0.485). However, we did not observe any significant correlation between the protein levels measured at admission and patient age.

### Auto- and cross-correlations of protein-level-time-series with a lag of 2 days

To assess relations between protein levels measured with a time difference of two days, we computed the auto- and the cross-correlations between the time series on days 1, 3, and 5 and the time series on days 3, 5, and 7 of all possible pairs of measured protein levels. The relevant coefficients are shown in Table 3.

**Table 3:** Auto- and cross-correlation matrix.

	MMP1	MMP2	MMP7	TIMP1	TIMP2	TIMP3	TIMP4
MMP1	**0.827** *p = 2*.*41∙10*^*−12*^	0.206 *p = 0*.*174*	0.223 *p = 0*.*141*	0.221 *p = 0*.*144*	0.223 *p = 0*.*140*	**0.450** *p = 0*.*0019*	0.009 *p = 0*.*953*
MMP2	-0.033 *p = 0*.*828*	-0.184 *p = 0*.*225*	-0.011 *p = 0*.*945*	-0.113 *p = 0*.*460*	-0.121 *p = 0*.*427*	-0.123 *p = 0*.*420*	-0.062 *p = 0*.*687*
MMP7	**0.350** *p = 0*.*018*	0.225 *p = 0*.*138*	0.285 *p = 0*.*058*	0.292 *p = 0*.*051*	0.235 *p = 0*.*120*	0.268 *p = 0*.*076*	0.281 *p = 0*.*062*
TIMP1	-0.152 *p = 0*.*319*	0.081 *p = 0*.*598*	**-0.369** *p = 0*.*013*	-0.059 *p = 0*.*700*	-0.075 *p = 0*.*623*	-0.239 *p = 0*.*114*	-0.074 *p = 0*.*628*
TIMP2	0.108 *p = 0*.*482*	-0.041 *p = 0*.*791*	0.063 *p = 0*.*683*	-0.197 *p = 0*.*195*	-0.109 *p = 0*.*476*	-0.084 *p = 0*.*585*	-0.121 *p = 0*.*430*
TIMP3	**0.572** *p = 3*.*99∙10*^*−5*^	**0.319** *p = 0*.*033*	0.294 *p = 0*.*050*	0.073 *p = 0*.*632*	0.159 *p = 0*.*298*	**0.304** *p = 0*.*043*	-0.179 *p = 0*.*239*
TIMP4	-0.234 *p = 0*.*123*	-0.016 *p = 0*.*915*	-0.238 *p = 0*.*115*	-0.252 *p = 0*.*095*	-0.285 *p = 0*.*058*	-**0.419** *p = 0*.*004*	*-0*.*116* *p = 0*.*448*

Autocorrelations are presented in the primary diagonal (gray cells). The autocorrelation represents the correlation of each variable on days 1–5 with later measurements of the same variable (days 3–7). Off-diagonal entries represent the average within-subject correlation between two variables measured on the same day.

### Univariable logistic analyses

Since one-third of the fatalities had already died on day 1 after the polytrauma, we performed univariable regression analyses with the day 0 level of each selected protein as the independent and the in-hospital mortality as the dependent variable. To meet the wide range of individual serum levels, we divided them by 1000 as the first step, meaning each OR refers to a difference of 1000 units (pg/mL). The results of the univariable logistic regression analyses are displayed in Table 4.

**Table 4:** Univariable logistic regression of every protein at admission.

Predictor	OR	95% CI		p-value
		lower	upper	
MMP1 day 0 x 10^−3^	0.820	0.548	1.228	0.335
MMP2 day 0 x 10^−3^	0.999	0.994	1.004	0.649
MMP7 day 0 x 10^−3^	0.981	0.817	1.178	0.838
TIMP1 day 0 x 10^−3^	0.989	0.979	0.99954	**0.041**
TIMP2 day 0 x 10^−3^	0.986	0.956	1.017	0.365
TIMP3 day 0 x 10^−3^	0.919	0.833	1.014	0.093
TIMP4 day 0 x 10^−3^	0.761	0.330	1.751	0.520

Since the TIMP1 day 0 level ranged from 55271 to 394953 pg/mL, we also calculated the OR for a difference of 20000 units to better interpret the result, obtaining an OR of 0.803 (95% CI = 0.650−0.991; p = 0.041).

### ROC statistics

**Fig 8:**
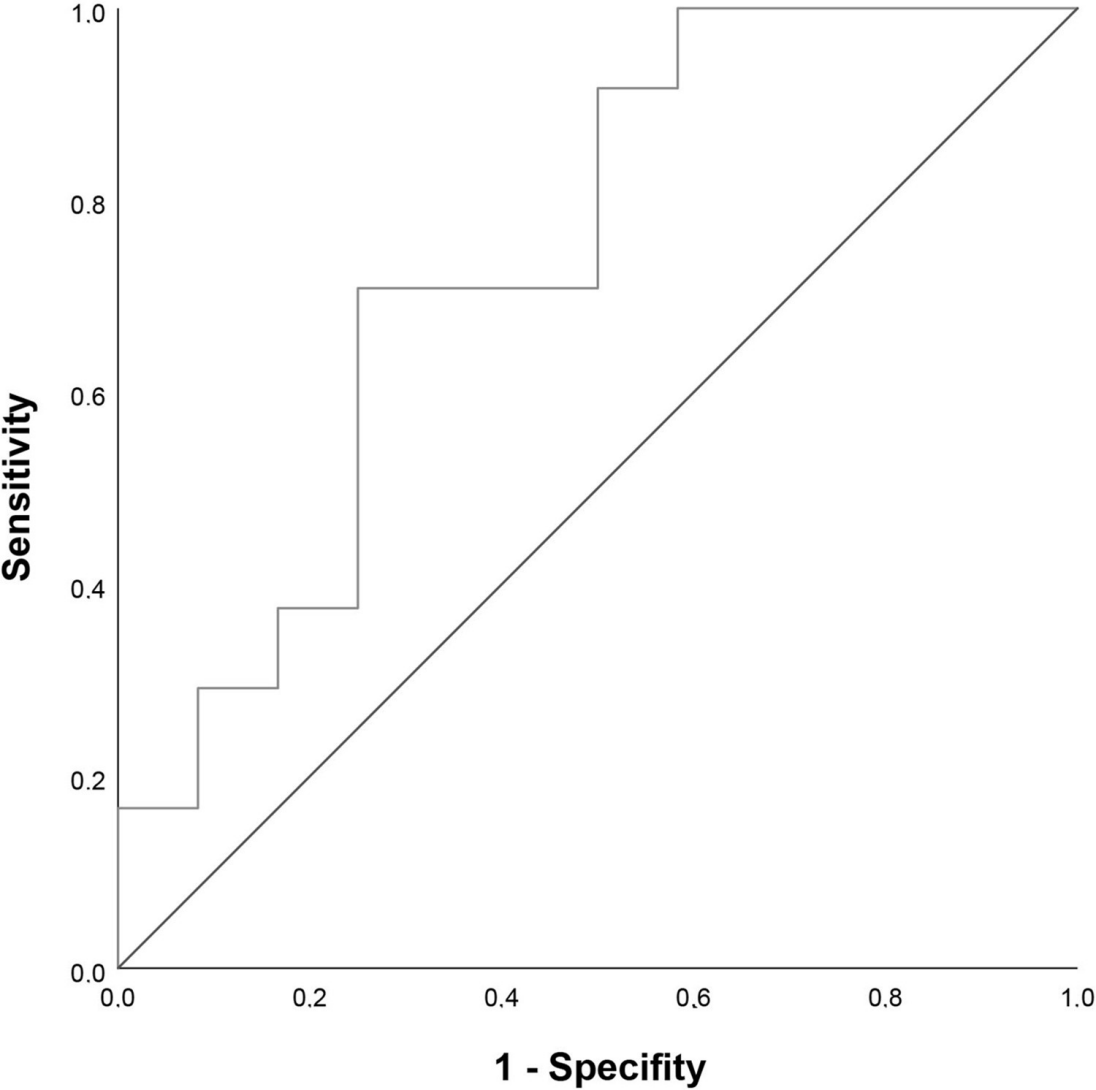
ROC curve for TIMP1 serum levels assessed at admission.

To illustrate the day 0 TIMP 1 level’s ability to distinguish between patients at high risk and low risk of dying, we plotted a ROC curve (Fig 8). It provided a cutoff level of 166488 pg/mL (sensitivity, 0.708; specificity, 0.750) and an AUC = 0.740, 95%CI [0.554, 0.925].

### Boxplots

**Fig 9:**
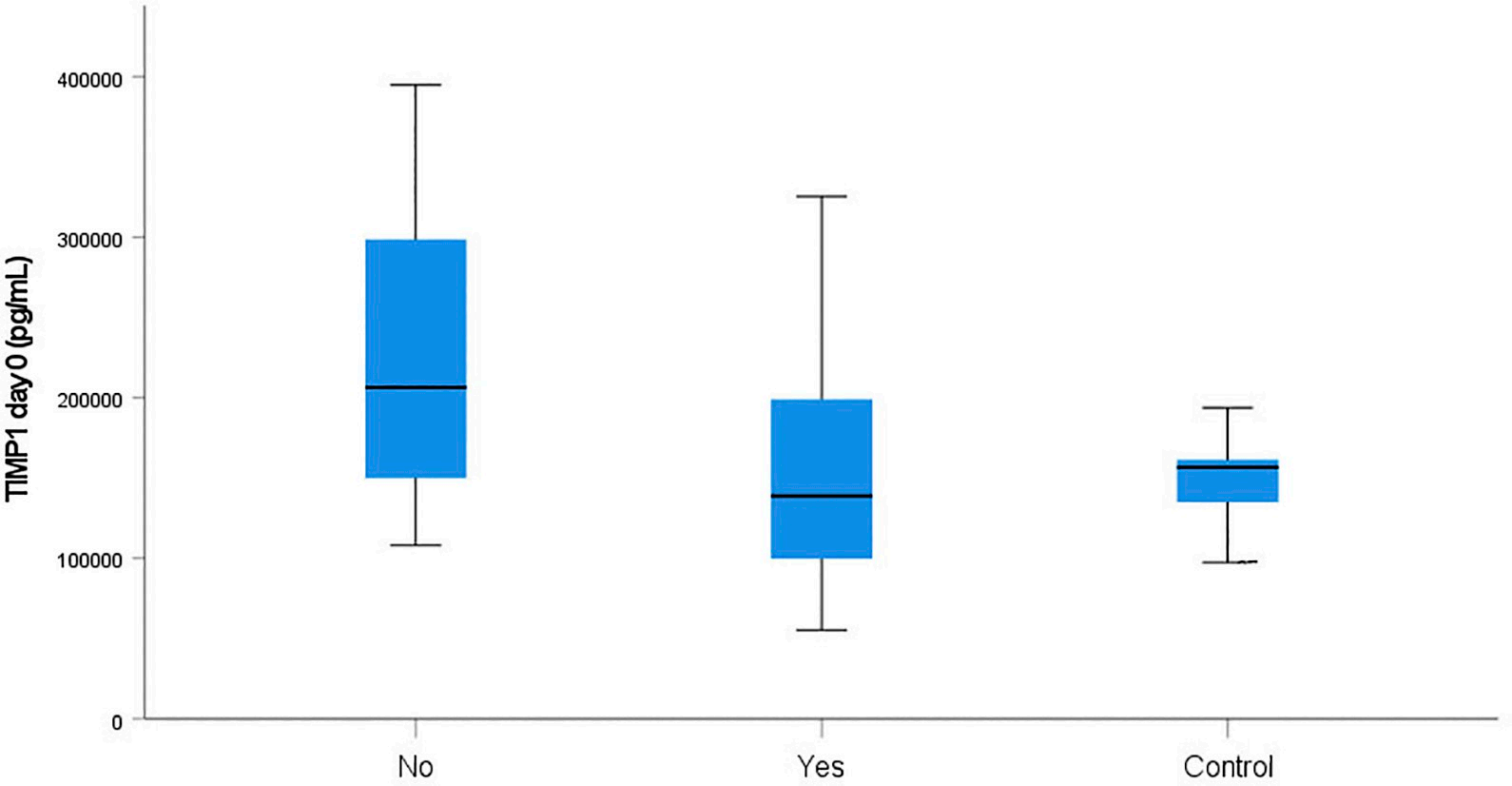
Distribution of TIMP1 serum levels. The boxplots display the TIMP1 levels of fatalities (Yes) and survivors (No) at admission in the study group and the control group.

At admission, median TIMP1 levels were significantly lower in fatalities than in survivors (138815 [55271−325486] pg/mL versus 207443 [108044−394953] pg/mL, p = 0.020). The relevant boxplots and the boxplot of the control group are displayed in Fig 9.

### Serum level ratios

We computed the median serum level quotients of significant MMP and TIMP pairs based on Spearman or cross-correlation over the study period (see Table 5).

**Table 5:** Median quotient of selected serum levels pairs.

	day 0	day 1	day 3	day 5	day 7	day 10
MMP2/TIMP2	2.235	2.499	2.751	2.729	2.938	2.690
MMP7/TIMP1	0.012	0.009	0.011	0.012	0.013	0.009
MMP1/TIMP3	0.121	0.118	0.167	0.242	0.296	0.066
MMP2/TIMP3	11.385	10.112	12.974	8.954	9.516	7.301

Furthermore, we conducted univariable logistic regression analyses, using each of the selected day 0 MMP/TIMP ratios as the independent, and death as the dependent variable. The results are presented in Table 6.

**Table 6:** Univariable logistic regression of MMP/TIMP ratios at admission.

Predictor	OR	95% CI		p-value
		lower	upper	
MMP2/TIMP2 day 0	0.789	0.341	1.826	0.580
MMP7/TIMP1 day 0	3.752 x e^15^	0.000	7.594 x e^40^	0.228
MMP1/TIMP3 day 0	79.216	0.008	754051.3	0.350
MMP2/TIMP3 day 0	1.121	0.947	1.326	0.184

## Discussion

The serum levels of the seven investigated proteins differed widely between individuals, resulting in similar trend curves of the median serum levels for TIMP1 and TIMP4 and for MMP1, MMP2, TIMP2, and TIMP3. Between MMP2, TIMP2, and TIMP3 levels, we also revealed a significant interrelationship at the same measurement points. For a two-day lag, we calculated a significant autocorrelation for MMP1 and TIMP3. Cross-correlations were significant between (1) MMP7 and MMP1, (2) TIMP1 and MMP7, (3) TIMP3 and MMP1, (4) TIMP3 and MMP2, and (5) TIMP4 and TIMP3 and almost significant between MM7 and TIMP1. Finally, the TIMP1 serum levels assessed at admission were associated with in-hospital mortality.

To our knowledge, we were the first to provide time trajectories, interrelationships, auto-correlations, and cross-correlations among the seven selected proteins in polytraumatized patients. Whereas the medians of MMP7 and TIMP1 exceeded the maximal reference value provided by the healthy controls starting with days 0 and 1, respectively, the medians of MMP1, MMP2, TIMP2, TIMP3, and TIMP4 remained within the relevant reference range. However, focusing on both individual and median serum levels did not provide evidence of a biomarker candidate to indicate treatment course in polytraumatized patients.

The moderate to very strong Spearman correlations between each two of the MMP2, TIMP2, and TIMP3 serum levels (Table 2) might indicate that these proteins were released by the same cells in large part, triggered by type and localization of the injuries. We used within-patient cross-correlations to determine if two proteins are "causative" related. Since the effect of an impact occurs with a delay, we computed the coefficients of repeated measurements between the time series of one protein level (assessed on days 1, 3, and 5) and the two-day-lagged version of the time series of another protein level (evaluated on days 3, 5, and 7). In Table 3, significant positive cross-correlation coefficients are displayed between MMP7 and MMP1, TIMP1 and MMP7, TIMP3 and MMP1, and TIMP3 and MMP2. These findings indicate that a rise in the first-mentioned protein level in a patient contributes to a rise in the second-mentioned two days later. On the other hand, an increase in the TIMP4 level adds to a time-delayed decrease in the TIMP3 level, as revealed by the significant negative cross-correlation coefficient. We focused on the ratios MMP2/TIMP2, MMP7/TIMP1, MMP1/TIMP3, and MMP2/TIMP3 due to significant Spearman or cross-correlations between pairs. However, neither time course nor univariable logistic regression analyses provided any evidence for clinical application.

In our study, autocorrelation refers to the repeated measurement correlation of a time series with its two-day-lagged version, representing their similarity. Table 3 presents significant autocorrelation coefficients for MMP1 (0.827) and TIMP3 (0.304), implying that the time series levels tend to follow the same direction. This issue is evident especially in Fig 1, since most individual trendlines are rising steadily.

Univariable binary regression analysis revealed a significant association between the TIMP1 level assessed at admission and in-hospital mortality. We calculated an OR of 0.803 for a difference of 20000 units, indicating that any increase of the TIMP1 level by 20000 pg/mL decreases the odds of dying by 19.7%. As an overall summary of the diagnostic accuracy of the TIMP1 day 0 level, we calculated the AUC. Unfortunately, 0.740, 95% CI = 0.554−0.925, is considered not clinically useful in general [31]. Therefore, if at all, TIMP1 is only suitable as a constituent of a biomarker panel that covers multiple pathways to identify polytraumatized patients in extremis.

A literature search has revealed a few studies that had already focused on TIMP1 and mortality in a trauma setting. Unfortunately, a meaningful comparison of results is impossible due to different study populations and non-compliance of endpoints in referenced papers with our in-hospital mortality assessment. Group comparison of survivors (>90-day survival) and non-survivors revealed lower mean TIMP1 serum levels in the fatalities within the first posttraumatic 24 hours [77]. In individuals who experienced severe blunt trauma (ISS ≥ 16; age ≥ 18 years), TIMP1 expression measured within the first 24 hours following the injury was found to have a significant correlation with survival at 90 days post-injury. Patients with a TBI (ISS 36 ± 14, age ≥ 18 years) had a significantly higher mean TIMP1 expression level within 72 hours of injury compared to those without intracranial lesions [78]. In patients suffering severe TBI (Glasgow Coma Scale < 9; ISS ≤ 9 in non-cranial aspects; age ≥ 18 years), serum TIMP1 levels assessed at the time of TBI occurrence were higher in fatalities than 30-day survivors and could predict 30-day mortality (OR = 1.01) [69]. Moreover, in a larger study population comprising these trauma victims, serum TIMP-1 levels were significantly higher during the first week of severe TBI in the non-surviving patients compared to those who survived for 30 days [79].

Since higher serum TIMP1 levels were associated with a lower mortality rate in our study group and the median TIMP1 level was higher in survivors and lower in fatalities compared to the control group (Fig 9), administering recombinant TIMP1 at hospital admission might reduce the risk of dying in polytraumatized patients. Another option might be to increase the production of endogenous TIMP1 by boosting serum MMP7 levels, as we revealed a nearly significant cross-correlation between MMP7 and TIMP1. Animal studies support our assumption that TIMP1 therapy might be beneficial. In mice, administration of TIMP1 has been shown to control acute bleeding in an experimental tail-hemorrhage model [80] and attenuate TBI-induced blood-brain barrier disruption in an experimental TBI model [81]. Moreover, according to data provided by neuronal culture models and in vivo models of TBI treatment with TIMP1, it significantly decreased neuronal death in mice [82].

To conclude, TIMP1 serum levels have already been measured in patients who suffered multiple injuries (ISS > 16) at various time points up to 72 hours post-trauma. The studies have indicated an increase in the average TIMP1 level between admission and 48 hours later, followed by a decrease between 48 and 72 hours [73, 77]. These results are in line with our findings (Fig 4).

Our study has a few limitations. Firstly, instead of performing an a priori power analysis, we based our sample size on the number of patients reported in published pilot studies [83–88]. Secondly, we could not provide a complete data set of serum protein levels for all survivors since the patient’s willingness determined sampling. Thirdly, our data were only suitable for studying univariable effects, and did not support multivariable regression analysis. Lastly, the study population was recruited only in one trauma center.

## Conclusions

The interrelationships and causal effects between some of the selected proteins might provide new insights into MMPs’ and TIMPs’ interactions. Exploring underlying causes may aid in identifying personalized therapies for multiple injured patients. Administering recombinant TIMP1 or increasing endogenous production might improve outcomes in polytrauma victims. However, before further investigations regarding basic research and clinical relevance are justified, our findings must be validated in a multicenter study using independent cohorts to account for clinical and biological variability.

## Supporting information

S1 Table Data underlying the findings. (XLSX)
